# The Effect of 13 Weeks Long-Distance Bicycle Riding on Inflammatory Response Indicators Related to Joint Cartilage and Muscle Damage

**DOI:** 10.3390/ijerph192316314

**Published:** 2022-12-06

**Authors:** Hyung-Jun Kim, Hyo-Cheol Lee

**Affiliations:** 1 Center for Sport Science in Chungnam, Asan-si 31580, Republic of Korea; 2 Department of Health and Sport Science, Korea National Sport University, Seoul 05541, Republic of Korea

**Keywords:** long-distance bicycle riding, inflammatory response, joint cartilage, muscle damage

## Abstract

This study was to investigate the effects of 13 weeks of long-distance cycling on biomarkers of joint cartilage, muscle damage and inflammation. All subjects in this study were seven participants of the “One Korea New-Eurasia Peace Cycle Expedition”, in which they rode cycles from Berlin, Germany to Seoul, Korea for thirteen weeks. The total course of the expedition was divided into three sub-courses: course 1 (from Berlin to Moscow), course 2 (from Moscow to Ulaanbaatar) and course 3 (from Ulaanbaatar to Seoul). All the selected participants rode 87.4 km/day (course 1), 70.4 km/day (course 2) and 57.6 km/day (course 3) on average, respectively. We collected their blood samples before the expedition in Seoul (S1), after course 1 in Moscow (M), after course 2 in Ulaanbaatar (U) and after the expedition in Seoul (S2), to analyze biomarkers of joint cartilage damage (Cartilage Oligomeric Matrix Protein; COMP), muscle damage (Creatine Phosphokinase; CPK, Lactate Dehydrogenase; LDH, Myoglobin), inflammation (Interleukin-6; IL-6, Interleukin-1β; IL-1β, Tumor Necrosis Factor-α; TNF-α, C-Reactive Protein; CRP) and stress hormone (Cortisol). According to this result, COMP (S1; 188.37 ± 46.68 ng/mL) showed a significant increase after the expedition course 1 (M; 246.69 ± 51.69 ng/mL, *p* = 0.012) and course 2 (U; 237.09 ± 62.57 ng/mL, *p* = 0.047), and recovered to the stable state after expedition course 3 (S2; 218.46 ± 34.78. *p* = 0.047). Biomarkers of muscle damage (CPK, LDH and Myoglobin) and inflammation (IL-6, IL-1β, TNF-α and CRP) were not significantly changed in all courses, but CRP (S1; 1.07 ± 0.76 ng/mL) showed a tendency to decrease after the expedition course 1 (M; 0.3 ± 0.1 mg/mL, *p* = 0.044). Lastly, the Cortisol level significantly increased in all courses (per *p* < 0.05), but the Cortisol level after expedition course 3 (S2; 21.00 ± 3.65 mg/mL) was lower than that of after the expedition course 1 (M; 24.23 ± 2.47 mg/mL, *p* = 0.028). In summary, it seems that repetitive and continuous 50–90 km/day of cycling can increase joint cartilage damage risk and stress hormone temporarily. However, this result suggests that the appropriate intensity of cycling for thirteen weeks does not increase physical damage, and rather enhances the human body to adapt to exercise training.

## 1. Introduction

Endurance training improves cardiopulmonary endurance and immune function, and is also effective in preventing various metabolic diseases, such as obesity, diabetes and cardiovascular disease. Given these benefits, more people are participating in endurance exercise [1,2], but long-distance endurance training without proper preparation can increase the probability of sudden death, and can also cause acute liver damage, muscle damage, bone and joint damage, hematuria, dehydration and gastrointestinal disease [3,4]. Thus, the safety of participation in long-distance endurance competition and sports should be investigated.

Cartilage oligomeric matrix protein (COMP) is a non-collagenous extracellular matrix protein that maintains collagen structure in joint cartilage. It is secreted into the blood when joint cartilage is damaged, and is thus used as an index of joint cartilage damage [5,6]. In other words, the influence of long-distance endurance training on indicators of joint cartilage damage may differ depending on exercise method, intensity and duration. Moreover, research has been lacking on the influence of exercises in which the effects of bodyweight on joints are limited, such as cycling or swimming, on joint cartilage damage. Córdova et al. (2015) collected blood samples from athletes before and after a 3-day cycling competition (123 km for day 1, 128 km for day 2 and 100 km for day 3) to measure serum CPK, myoglobin and LDH concentrations; the authors found that CPK did not change significantly after the competition on day 1 and before the competition on day 2, but significantly increased after the competition on day 2 until recovery day 1 after the competition on day 3 [7]. Furthermore, Kim et al. (2009) showed that the concentration significantly increased up to 4 days after the completion of the 100–200 km ultramarathon [8]. In contrast, serum myoglobin concentration increased significantly after each competition, but returned to normal before the competition on the next day. Cytokines are regulatory proteins secreted by cells in response to various stimuli, which act as major regulatory factors in various aspects, such as immune response, muscle atrophy and development, insulin sensitivity and fat metabolism [9,10,11]. Interleukin 6 (IL-6) and 1 beta (IL-1β), which are pro-inflammatory cytokines, and tumor necrosis factor alpha (TNF-α), which is an inflammatory cytokine, are major indicators of muscle damage and inflammatory response [12,13]. IL-6 is known to be involved in various responses associated with inflammation and damage, including its role in increasing cortisol and C-reactive protein (CRP), another inflammatory marker [14]. Chiu et al. (2015) reported that in the concentration of IL-6 and TNF- α, CRP was significantly increased from immediately after exercise to 24 h after exercise in 24 men who completed a 100 km ultramarathon [15]. Although one-time long-distance endurance exercise has been reported to increase the serum concentrations of IL-6, TNF-α and CRP [12,16,17], changes in inflammatory markers with long-distance exercise still remain controversial.

In summary, with increases in the participation of non-professional athletes in endurance exercise, as well as increases in competitions and sports events, it is important to elucidate the influence of long-distance endurance exercise on markers of tissue damage in the body, in order to set safe exercise plans. Nevertheless, the influence of long-distance endurance exercise on joint cartilage damage, muscle damage and level of inflammation according to exercise type, distance and duration remains unclear, with a lack of relevant research. Therefore, this study aimed to investigate the influence of 13 weeks of long-distance cycling on inflammatory markers associated with muscle and joint cartilage damage.

## 2. Materials and Methods

### 2.1. Study Participants

**Figure 1:**
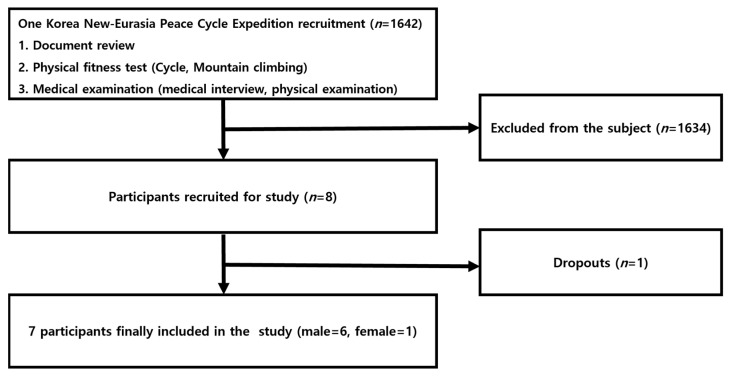
The participant recruitment and selection process.

The participants of this study were adults aged from in their 20s to their 50s who did not have any musculoskeletal disorder, but had more than 7 years of experience with cycling. Furthermore, they were healthy individuals who had not participated in an exercise program or diet for the 3 months, and no specific disease was found in the medical examination. The participants were screened for their cycling abilities to complete the ultra-long-distance cycling event across Europe and Asia (One Korea New-Eurasia Peace Cycle Expedition, Figure 1). The participants consisted of 6 male and 1 female cyclist selected for the expedition (Table 1). The selected participants all received detailed explanation of the study purpose and procedures, and provided written consent. Ethical permission was granted from the Korea National Sport University’s Ethics Committee (Korea National Sport University Industry-Academic Cooperation Foundation-985), and all procedures were in accordance with the Declaration of Helsinki for research on human subjects.

### 2.2. Study Design and Participants

#### 2.2.1. Cycling

**Figure 2:**
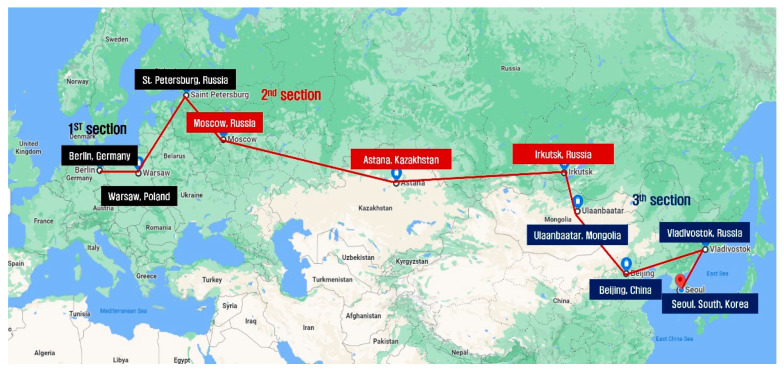
The course of One Korea New-Eurasia Peace Cycle Expedition. 1st section, from Berlin to Moscow (black square), 2nd section, from Moscow to Ulaanbaatar (red square) and 3rd section, From Ulaanbaatar to Seoul (blue square).

According to the program of the One Korea New-Eurasia Peace Cycle Expedition, the participants cycled 4869 km from the 15,000 km-long expedition. The total course of the expedition was divided into three sub-courses: course 1 (from Berlin to Moscow); course 2 (from Moscow to Ulaanbaatar); and course 3 (from Ulaanbaatar to Seoul). Although the original plan was to cycle for 5 days and have 1 day of rest, some changes were made depending on environmental conditions (weather, route conditions and cycling permissions). Figure 2 (Google map) and Table 2 show the sub-courses and cycling content.

#### 2.2.2. Time and Method for Sample Collection

Blood samples were collected in the morning of the day after cycling after fasting at the following time points: before the expedition (in Seoul); during the expedition in Moscow (day 29) and Ulaanbaatar (day 63); and on the last day of expedition in Seoul (day 93). The collected samples were immediately placed into serum tubes and EDTA tubes and centrifuged (Beckman, Brea, CA, USA, 15 min/3000 rpm). The supernatant serum and plasma were separated and carried on dry ice (−78.5 °C), and stored at −80 °C in a freezer until analysis.

#### 2.2.3. Sample Analysis

In order to assess the level of joint cartilage and muscle damage from long-distance cycling, the concentration of COMP, an index of joint cartilage damage, was measured using Human COMP Quantikine ELISA kits (R&D systems Minneapolis, Minneapolis, MN, USA) through an enzyme-linked immunosorbent assay (ELISA). The levels of CPK, LDH and myoglobin, which are markers of muscle damage, were analyzed using Modular Analytics (Roche, Mannheim, Germany) through UV assay. Inflammatory markers (IL-6, IL-1β and TNF-α) were analyzed using Human High Sensitivity Cytokine kits (R&D systems Minneapolis, Minneapolis, MN, USA) through Luminex assay, and CRP was analyzed using Modular Analytics (Roche, Germany) through immunoturbidimetric assay. The concentration of cortisol, a stress hormone, was analyzed using a fully automated chemistry analyzer (Beckman, Brea, CA, USA). This study was conducted as a joint study. The results pertaining to serum cortisol have already been published through Gil’s study [18]. However, because the results were obtained from the same participants and because evidence for the influence of this study’s exercise type on tissue damage response was needed, the results have been cited in this study as well.

#### 2.2.4. Data Analysis

Mean and standard deviation were calculated for the collected data using SPSS/PC+Version 20.0. One-way repeated measures ANOVA was used to assess the differences in serum markers according to cycling distance. When differences were present, they were analyzed through Duncan multiple test analysis, and the level of statistical significance was set as *p* < 0.05.

## 3. Results

### 3.1. Changes in Markers of Joint Cartilage Damage

**Figure 3:**
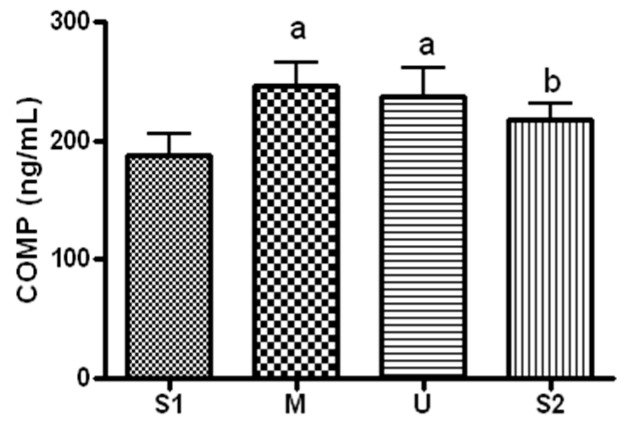
Serum COMP concentration in different locations. Values are mean ± S.D. Significance (*p* < 0.05) is denoted by a: relative to S1 and b: relative to M. S1: Before start in Seoul, M: Moscow U: Ulaanbaatar S2: Return to Seoul.

When serum COMP concentration, which is an indicator of joint cartilage damage, was analyzed (Figure 3), the concentration was 188.37 ± 46.68 ng/mL before the expedition, 246.69 ± 51.69 ng/mL after course 1 (Berlin–Moscow), 237.09 ± 62.57 ng/mL after course 2 (Moscow–Ulaanbaatar) and 218.46 ± 34.78 ng/mL after course 3 (Ulaanbaatar–Seoul). Compared to pre-expedition value, the concentration increased after course 1 (*p* = 0.012) and course 2 (*p* = 0.047). Compared to course 1, the concentration decreased after course 3 (*p* = 0.036).

### 3.2. Changes in Markers of Muscle Damage

#### 3.2.1. Changes in CPK

**Figure 4:**
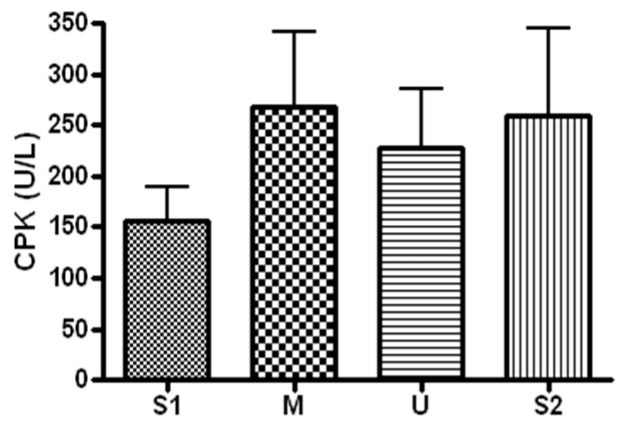
Serum CPK concentration in different locations. Values are mean ± S.D. S1: Before start in Seoul, M: Moscow U: Ulaanbaatar S2: Return to Seoul.

When serum CPK concentration, a marker of muscle damage, was analyzed (Figure 4), the concentration was 156 ± 85.84 U/L before the expedition, 267.28 ± 197.66 U/L after course 1 (Berlin–Moscow), 227.71 ± 155.22 U/L after course 2 (Moscow–Ulaanbaatar) and 259.57 ± 227.94 U/L after course 3 (Ulaanbaatar–Seoul). There was no significant difference noted at subsequent measurements compared to the baseline (*p* > 0.05).

#### 3.2.2. Changes in Myoglobin

**Figure 5:**
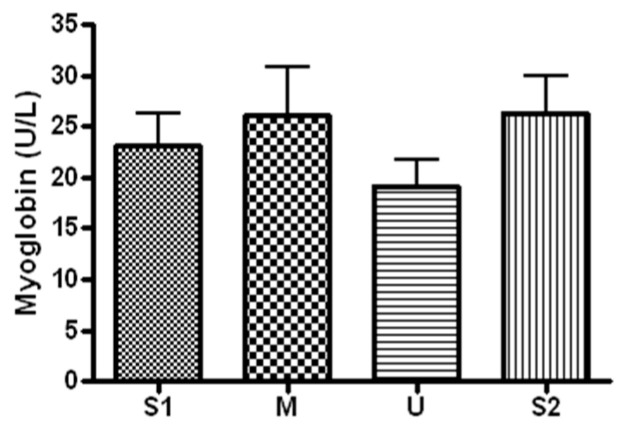
Serum myoglobin concentration in different locations. Values are mean ± S.D. S1: Before start in Seoul, M: Moscow U: Ulaanbaatar S2: Return to Seoul.

When serum concentration of myoglobin, an indicator of muscle damage, was analyzed (Figure 5), the concentration was 23.14 ± 8.49 U/L before the expedition, 26.05 ± 12.83 U/L after course 1 (Berlin–Moscow), 19.17 ± 6.56 U/L after course 2 (Moscow–Ulaanbaatar) and 26.35 ± 9.71 U/L after course 3 (Ulaanbaatar–Seoul). The concentration did not differ significantly from the baseline at each of the subsequent measurements (*p* > 0.05).

#### 3.2.3. Changes in LDH

**Figure 6:**
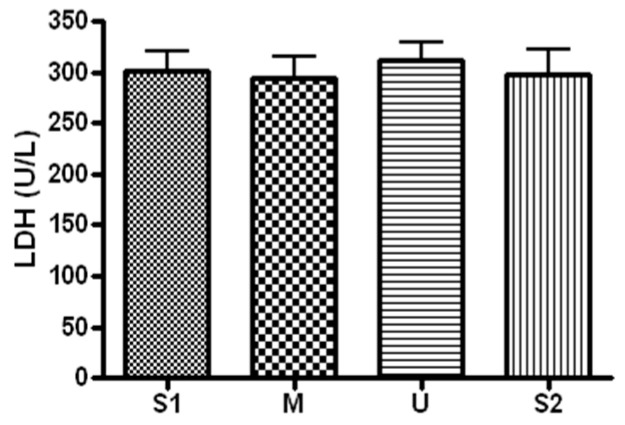
Serum LDH concentration in different locations. Values are mean ± S.D. S1: Before start in Seoul, M: Moscow U: Ulaanbaatar S2: Return to Seoul.

When serum concentration of LDH, an index of muscle damage, was analyzed (Figure 6), the concentration was 301.71 ± 49.21 before the expedition, 294.57 ± 54.93 U/L after course 1 (Berlin–Moscow), 311.28 ± 45.45 U/L after course 2 (Moscow–Ulaanbaatar) and 298.42 ± 62.83 U/L after course 3 (Ulaanbaatar–Seoul). There was no significant difference noted at subsequent measurements compared to the baseline (*p* > 0.05).

### 3.3. Changes in Inflammatory Markers

#### 3.3.1. Changes in IL-6

**Figure 7:**
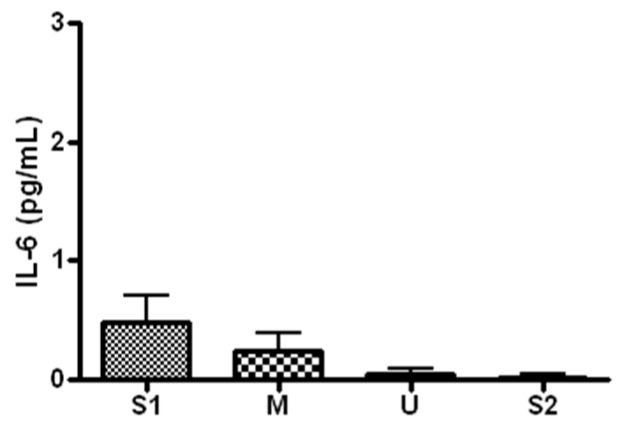
Serum IL-6 concentration in different locations. Values are mean ± S.D. S1: Before start in Seoul, M: Moscow U: Ulaanbaatar S2: Return to Seoul.

When serum concentration of IL-6, an inflammatory marker, was analyzed (Figure 7), the concentration was 0.62 ± 0.44 pg/mL before the expedition, 0.37 ± 0.17 pg/mL after course 1 (Berlin–Moscow), 0.06 ± 0.09 pg/mL after course 2 (Moscow–Ulaanbaatar) and 0.04 ± 0.05 pg/mL after course 3 (Ulaanbaatar–Seoul). The concentration did not differ significantly from the baseline at each of the subsequent measurements (*p* > 0.05).

#### 3.3.2. Changes in IL-1β

**Figure 8:**
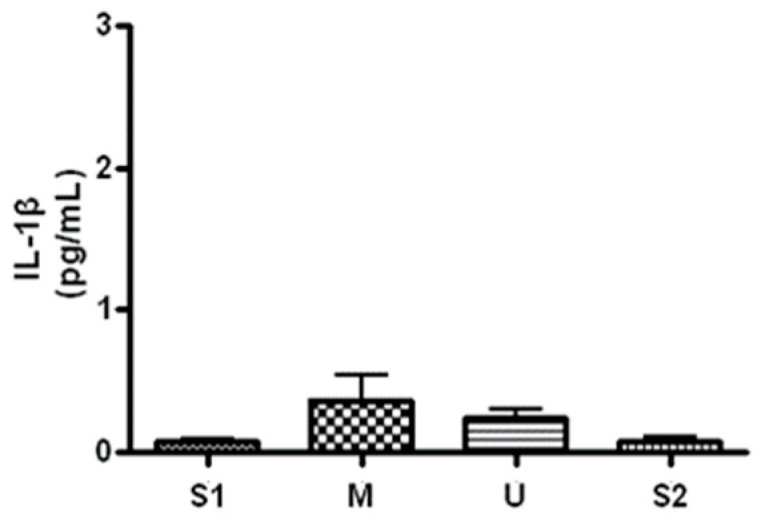
Serum IL-1β concentration in different locations. Values are mean ± S.D. S1: Before start in Seoul, M: Moscow U: Ulaanbaatar S2: Return to Seoul.

When serum concentration of IL-1β, an inflammatory marker, was analyzed (Figure 8), the concentration was 0.07 ± 0.02 pg/mL before the expedition, 0.36 ± 0.30 pg/mL after course 1 (Berlin–Moscow), 0.23 ± 0.11 pg/mL after course 2 (Moscow–Ulaanbaatar) and 0.07 ± 0.06 pg/mL after course 3 (Ulaanbaatar–Seoul). There was no significant difference noted at subsequent measurements compared to the baseline (*p* > 0.05).

#### 3.3.3. Changes in TNF-α

**Figure 9:**
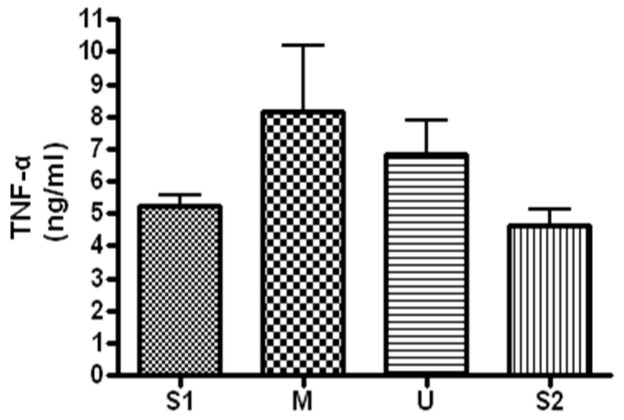
Serum TNF-α concentration in different locations. Values are mean ± S.D. S1: Before start in Seoul, M: Moscow U: Ulaanbaatar S2: Return to Seoul.

When serum concentration of TNF-α, an inflammatory marker, was analyzed (Figure 9), the concentration was 5.25 ± 0.78 ng/mL before the expedition, 8.16 ± 4.89 ng/mL after course 1 (Berlin–Moscow), 6.85 ± 2.56 ng/mL after course 2 (Moscow–Ulaanbaatar) and 4.61 ± 1.3 ng/mL after course 3 (Ulaanbaatar–Seoul). There was no significant difference noted at subsequent measurements compared to the baseline (*p* > 0.05).

#### 3.3.4. Changes in CRP

**Figure 10:**
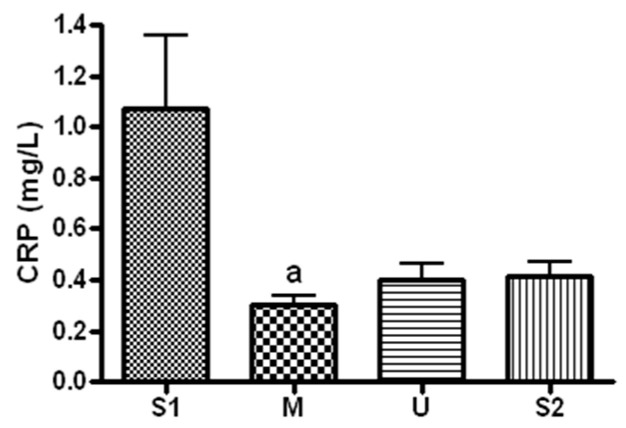
Serum CRP concentration in different locations. Values are mean ± S.D. Significance (*p* < 0.05) is denoted by a: relative to S1. S1: Before start in Seoul, M: Moscow U: Ulaanbaatar S2: Return to Seoul.

When serum concentration of CRP, an inflammatory marker, was analyzed (Figure 10), the concentration was 1.07 ± 0.76 mg/mL before the expedition, 0.3 ± 0.1 mg/mL after course 1 (Berlin–Moscow), 0.4 ± 0.16 mg/mL after course 2 (Moscow–Ulaanbaatar) and 0.41 ± 0.14 mg/mL after course 3 (Ulaanbaatar–Seoul). Compared to the pre-expedition measurement, the concentration decreased significantly after course 1 (*p* = 0.044).

### 3.4. Changes in Stress Hormone

#### Changes in Cortisol

**Figure 11:**
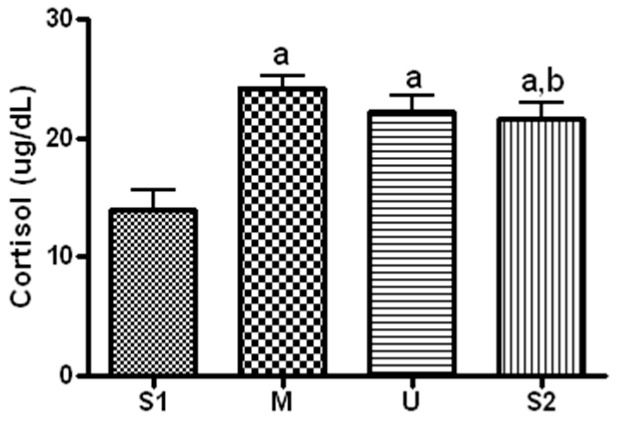
Serum Cortisol concentration in different locations. Values are mean ± S.D. Significance (*p* < 0.05) is denoted by a: Relative to S1 and b: Relative to M. S1: Before start in Seoul, M: Moscow U: Ulaanbaatar S2: Return to Seoul.

When serum concentration of cortisol, a stress hormone, was analyzed (Figure 11), the concentration was 13.92 ± 4.52 ug/dL before the expedition, 24.23 ± 2.47 ug/dL after course 1 (Berlin–Moscow), 22.18 ± 3.52 ug/dL after course 2 (Moscow–Ulaanbaatar) and 21 ± 3.65 mg/mL after course 3 (Ulaanbaatar–Seoul). Compared to the pre-expedition value, the values obtained from all of the subsequent measurements had significant increases (*p* < 0.05), and the concentration decreased significantly after course 3 compared to that after course 1 (*p* = 0.028).

## 4. Discussion

The present study was conducted to assess the influence of 13 weeks of long-distance cycling on serum markers of joint cartilage damage, muscle damage and inflammatory response after each of 3 sub-courses (course 1: Berlin–Moscow; course 2: Moscow–Ulaanbaatar; and course 3: Ulaanbaatar–Seoul). According to the results, joint cartilage damage and stress hormone increased in proportion to daily cycling distance in each course during the 13 weeks of long-distance cycling, and the values decreased with increases in cycling duration. Inflammatory response was found to decrease with long-distance cycling. In summary, although markers of damage had a positive correlation with daily cycling distance, with long-distance cycling, the participants adapted to the given exercise intensity, and had overall positive training effects in terms of muscle damage, joint cartilage damage and inflammatory response.

During the 13 weeks of long-distance cycling expedition, serum COMP concentration increased after course 1 and course 2. After course 3, the concentration returned to the normal range from the increased values measured after courses 1 and 2. The COMP concentration that increased after course 1 would have stayed elevated until after course 2, as the cycling distance was longer for courses 1 and 2 than course 3. Moreover, joint cartilage would have adapted to long-distance repeated exercise over the three courses. In this study, the participants cycled 87 km per day on average in course 1 for a total distance of 2097 km, 70 km per day on average in course 2 for a total of 1678 km and 57 km per day on average in course 3 for a total of 1094 km. Therefore, the daily cycling distance would have influenced the serum COMP concentration. Qi & Changlin (2006) measured COMP every 2 weeks after 10 weeks of exercise training in lab animals; the serum concentration was highest at weeks 4 and 6, and started to decrease from week 10 [19]. Corsetti et al. (2015) showed that the COMP of cyclists who rode a distance of 3500 km for 3 weeks decreased after 12 weeks [20]; this finding was explained by the fact that cartilage is capable of continuous regeneration. When cartilage damaged by an exercise stimulus regenerates, the regenerated cartilage is more resistant. Therefore, the improved resistance of cartilage at week 10 would have decreased joint damage from the exercise stimulus. Similarly, the decrease in serum COMP concentration after course 3 in the present study could be interpreted as adaptation of joint cartilage to long-distance cycling. However, since specific information on the influence of long-distance cycling on markers of joint cartilage damage has been lacking thus far, it may be difficult to generalize the present study’s findings.

High-intensity exercise is known to increase the serum concentration of muscle damage markers, and such changes are influenced by exercise intensity, duration, type and temperature during exercise [13,21,22,23,24]. In the present study, 13 weeks of long-distance cycling did not have any significant influence on the expression of serum CPK, LDH and myoglobin. Previous studies have reported that muscle damage markers are influenced by the participants’ exercise experience. In particular, trained athletes have smaller increases in muscle damage markers after exercise compared to untrained individuals, and trained individuals also recover faster [22,25,26,27]. The participants of this study are experienced cyclists capable of ultra-long-distance cycling who have been selected through tests of cycling ability. The daily cycling distance in this study may not have influenced changes in muscle damage markers, as the participants already had improved muscle resistance and resilience. Fallon et al. (1999) measured CPK concentration before 16 days of 1600 km ultramarathon, during the ultramarathon (days 4 and 11) and after the ultramarathon, and reported that the concentration increased on day 4 but decreased on day 11, and after the ultramarathon [28]. Ehlers et al. (2002) measured serum concentration of CPK before and during 10 days of training of identical intensity (on days 4, 7 and 10) in college soccer players, and reported that the concentration increased most on day 4 and returned to normal on day 10 [29]. In Chen et al.’s study (2001), 22 college students performed eccentric exercise every day for 7 days [30]. When serum CPK and LDH concentrations were measured, the concentration increased most on days 3 and 4, but returned to the baseline on day 7. Previous studies interpreted that long-distance exercise decreases serum markers of muscle damage, which increase after short-term exercise. The decrease was attributed to improved resistance of muscles as a result of exercise training. Therefore, muscle damage markers would not have changed in the present study after 13 weeks of long-distance cycling for the following reasons. First, the daily cycling distance may have been insufficient to influence muscle damage in our well-trained participants. Second, muscles may have metabolically adapted to long-distance cycling, and this may have increased their resistance to exercise stimulus or increased the half-life of damage markers.

IL-6, IL-1β, TNF-α and CRP are well-known markers of inflammation. They increase in specific conditions, and indicate the level of inflammation in the muscles and body [12,13,14]. Although these inflammatory markers are reported to increase with short-term intense exercise [31,32], their response to various types of external stimuli, including exercise type, target, intensity and duration, still remains controversial [33,34]. In the present study, the concentration of IL-6, IL-1β and TNF-α did not differ from baseline at every subsequent measurement according to cycling distance during the 13 weeks of long-distance cycling. In contrast, serum CRP concentration decreased after course 1. Although the changes were not significant, the concentration also tended to decrease after courses 2 and 3 [35,36,37]. In Meyer et al.’s study (2006), 33 obese adolescents performed aerobic exercise for 6 months [38]. When inflammatory markers were measured in these adolescents, serum CRP concentration was found to have decreased. Similarly, Mattusch et al. (2000) had 12 runners perform 10–20 km of running training every week for 9 months, and reported decreases in serum CRP concentration [39]. Moreover, various studies analyzing the relationship between physical activity and serum CRP concentration reported that CRP was lower in individuals who are physically active [40,41,42]. Previous studies attribute such a decrease in CRP with long-distance exercise directly to decreased production of cytokines, and indirectly to improved insulin sensitivity and antioxidative activity [42,43,44,45,46]. The present study’s findings agree with previous research reports. In other words, daily cycling distance during the 13 weeks of long-distance cycling seems to have improved inflammatory response by causing positive changes in the body, such as improved immune function. From a clinical point of view, daily cycling exercise for more than 12 weeks will reduce inflammation-related factors, and long-distance cycling will be useful for recovery and rehabilitation.

Cortisol is a stress hormone secreted by the adrenal cortex in response to various stimuli, which is known to suppress adrenocorticotropic hormone, promote gluconeogenesis and mediate inflammatory responses [47]. When serum cortisol concentration was analyzed during long-distance cycling, the concentration increased from the baseline at all subsequent measurements. The increase was greatest after course 1, compared to which concentration decreased significantly after course 3. Cortisol increases in proportion to exercise duration and intensity [48]. Therefore, 13 weeks of long-distance cycling could increase serum stress hormone, and daily cycling distance during each sub-course would have influenced changes in serum cortisol. However, cortisol is influenced by various factors in addition to exercise. Therefore, more comprehensive studies are required to attribute the changes in serum cortisol to exercise.

Regarding the limitations of the present study, for the results of this study to show a moderate difference, 80* power and 5% significance level, 24 subjects are needed [49]. However, the statistical significance is weak due to the small number of subjects, and the results of the study cannot be generalized. Therefore, a follow up study with an expanded number of subjects should be conducted. Additionally, several factors could not be controlled during the long-distance cycling expedition due to the inherent characteristics of field studies. The subjects’ diet, nutrition and psychological status were not controlled, which may have affected weight gain. Furthermore, only limited information could be collected from a small number of participants (*n* = 7), and the direct and indirect influence of changing geographic characteristics and climatic changes during the expedition on the participants could not be excluded or quantified objectively.

## 5. Conclusions

After 13 weeks of long-distance cycling, joint cartilage damage markers and stress hormone increased in proportion to daily cycling distance during each sub-course, and decreased with increases in exercise duration. Inflammatory response decreased with long-distance cycling. The results suggest that continued cycling for 50–90 km per day temporarily increases joint cartilage damage and stress hormone in the initial phases of adaptation. However, continued (13 weeks) long-distance cycling of appropriate intensity decreases the damage response through adaptation of tissues to exercise training, rather than increasing body damage.

## Figures and Tables

**Table 1:** The characteristics of subjects.

No.	Sex	Age (Yr.)	Height (cm)	Fat (%)	S1 (kg)	M (kg)	U (kg)	S2 (kg)
1	Male	23.0	176.1	14.5	67.7	69.1	70.0	72.9
2	Male	51.6	174.0	22.1	79.7	81.0	83.5	81.0
3	Male	22.8	177.4	10.0	75.4	76.8	81.2	81.0
4	Male	45.1	174.0	17.7	71.8	73.8	74.3	73.9
5	Female	33.9	156.9	32.7	59.5	58.3	58.9	59.6
6	Male	29.7	180.5	14.4	73.4	72.2	75.8	79.0
7	Male	25.5	177.0	30.8	90.3	86.4	85.8	89.8

S1: Before start in Seoul, M: Moscow U: Ulaanbaatar S2: Return to Seoul.

**Table 2:** Cycling content.

	1st Section ^(1)^	2nd Section ^(2)^	3rd Section ^(3)^
Cycling distance (km)	2097	1678	1094
Total days	29	34	30
Cycling days	24	24	19
Mean cycling (km)	87.4	70	57.6
The longest cycling (km)	132	147	117
The shortest cycling (km)	20	14	10
Mean temperature (°F)	16.5	1.7	1.6
Maximum temperature (°F)	22	15	18
Minimum temperature (°F)	−1	−7	−7

^(1)^ From Berlin to Moscow, ^(2)^ From Moscow to Ulaanbaatar, ^(3)^ From Ulaanbaatar to Seoul.

## Data Availability

Not applicable.
